# A Case Report of Familial Facial Nerve Palsy in an Infant

**DOI:** 10.7759/cureus.101099

**Published:** 2026-01-08

**Authors:** Dallin Stevens, Steven J Laxton, Dexter Woods

**Affiliations:** 1 Emergency Medicine, University of Tennessee Health Science Center (UTHSC) - Nashville/Ascension Saint Thomas Rutherford, Murfreesboro, USA

**Keywords:** bells palsy, facial droop without forehead involvement, facial nerve palsy, facial palsy, infantile facial nerve palsy, juvenile facial nerve palsy

## Abstract

This is a case report of a three-year-old patient who was evaluated in the emergency department with noted facial nerve palsy in the setting of a family history of facial nerve palsy at similar ages. The patient presented with unilateral facial nerve palsy, with multiple family members experiencing this illness at young ages. Data is still insufficient to know the optimal treatment in this age group; however, following data for ages 10 and above, steroids and ocular lubricant were prescribed, and the patient was sent for follow up with their primary care physician and was referred to a neurologist. The toddler at three months follow up had complete resolution of the facial nerve palsy without complication.

## Introduction

The facial nerve, also known as the seventh cranial nerve (CN VII), is responsible for motor innervation of the muscles of facial expression [1]. In addition to motor functions, it carries parasympathetic fibers to lacrimal and salivary glands, as well as sensory and taste fibers from the anterior two‑thirds of the tongue and parts of the external ear [1]. A unique feature of the facial nerve is its formation in the brainstem. It travels posteriorly from the facial nucleus and courses around the abducens nucleus before exiting the lower part of the pons anterior-laterally. This unique feature can lead to facial nerve injury presentations that originate in the brainstem. Anatomically, it emerges from the brainstem, travels through the temporal bone’s facial canal, exits via the stylomastoid foramen, and then branches across the face. Its complex pathway comprises intracranial (cisternal to mastoid) and extracranial segments, critical for clinical and imaging assessments [2]. Understanding its anatomy is essential for diagnosing lesions or dysfunction caused by trauma, infection, inflammation, or tumors. In Bell's palsy, it is usually a peripheral nerve issue or an issue confined to the extracranial segment. There seems to be a correlation between age and the incidence of Bell’s palsy, as well as a previously documented but not clearly defined familial component. The incidence in patients younger than 10 years is 2.7/100,000, while in patients between 10 and 20 years old is approximately10.1/100,000 [3].

Bell’s palsy is an acute, idiopathic peripheral facial nerve (CN VII) palsy, resulting in sudden weakness or paralysis of one side of the face [4]. It typically develops rapidly, reaching maximal severity within 48-72 hours, and is a diagnosis of exclusion [5]. Although the exact cause remains uncertain, a reactivated herpes simplex virus infection involving nerve inflammation is the most commonly proposed mechanism of illness [6]. Other contributing factors may include ischemia, immune‑mediated inflammation, anatomical predisposition, or cold exposure. Most patients recover fully over weeks to months, although some may experience residual weakness or complications like synkinesis or corneal damage if not taking appropriate steps to maintain ocular lubrication.

Clinically, Bell’s palsy typically presents with a rapid-onset drooping of one side of the face, characterized by difficulty closing the eye, a flattened nasolabial fold, a drooping mouth corner, and inability to smile symmetrically [7]. Due to the unique nature of the facial nerve’s course it is imperative that the clinician evaluate the abducens nerve function by verifying intact lateral gaze. Paralysis of the ipsilateral abducens nerve should be cause for concern. This most likely represents an infarct in the region of the abducens nucleus. Patients may also experience drooling, pain behind the ear or jaw, altered taste, hyperacusis (sound sensitivity), headaches, and changes in tear or saliva production [7]. Some lose complete control of facial muscle movement on the affected side, giving the appearance of an expressionless, smooth half‑face. Symptoms begin suddenly and often peak within two to three days. While most improve within weeks to months, a minority may have lasting asymmetry or functional complications.

Although Bell’s palsy is idiopathic, the most widely accepted theory attributes it to reactivation of herpes simplex virus type 1 (HSV‑1), leading to inflammation and swelling of the facial nerve within the narrow facial canal. HSV‑1 has been detected in up to 50% of affected patients in some studies, with varicella‑zoster virus (another herpes family member) implicated in approximately 13% of cases [8]. This swelling can compress the nerve, restricting blood flow and impairing nerve function. Additional theories include vascular ischemia, autoimmune‑mediated inflammatory damage, structural anatomy, and environmental triggers like cold exposure. Among these, viral reactivation-especially HSV‑1-is the most consistently associated factor.

In previously published literature, a familial component has been described; however, it is infrequent, and very few reports exist. Most prior reports inlude the ages 10 years old and older, such as in one report where a family of four was noted to have experienced Bell’s Palsy. This affected them at younger ages, with family members having experienced it at ages five and nine, with other members at ages 13 and 15, 14, 19, 33, and 38, respectively [9], suggesting that there can exist a familial component to idiopathic facial nerve palsy. This report is novel in that it describes a case of extensive familial history of facial nerve palsy at a young age, with a new onset of facial nerve palsy at the young age of three years old.

## Case presentation

This is a three-year-old female with no past medical history, up to date with vaccines, who presented to the emergency department with a right-sided facial droop that started over the past day. The patient's mother stated that the last known normal was the night prior to presentation to the emergency department and that the patient woke up in the morning with a right-sided facial droop. She states that the patient has been acting like her normal self apart from the facial droop that was noted by family members. The patient's mother denies any extremity weakness, changes in movement, injuries, or head trauma. The patient also did not report sensory changes. She does have an established pediatrician that she follows regularly with no current medical conditions. The mother also reported no complications at birth. The mother reports that she has a family history of Bell’s palsy around a similar age in the patient’s father, along with a history of Bell’s palsy in three of the patient’s aunts, as well; however, they do not recall the exact age of occurrence. No further associated symptoms were reported.

Vital signs were measure at a temperature of 97.6°F, heart rate 102 beats per minute, respiratory rate of 26, blood pressure of 84/50, and oxygen saturation of 99% on room air. The patient's physical exam showed findings of right-sided facial droop involving the forehead, normal extra-ocular muscle movement (no abducens nerve involvement),with normal sensation to light touch over the face, upper and lower extremities. The patient also had normal strength in the upper and lower extremities. Given the clinical history of familial facial nerve palsy, imaging of the brain was not indicated at this time. Blood glucose was measured at 84 mg/dL.

Given the clinical history of familial facial nerve palsy, the patient was treated with prednisone 0.15 mg/kg once daily for five days as well as ocular lubricating eye drops with instructions to follow up with their pediatrician. A referral for pediatric neurology was also given. The patient's mother was contacted three months following presentation to the emergency department, and there was a reported complete resolution of the right facial nerve palsy without complication.

## Discussion

As previously discussed, the incidence of Bell’s palsy in patients older than 10 years is 2.7/100,000 [10]. Bell’s palsy can be disconcerting to both patients and health care providers because it is an acute nerve issue that also presents in a similar fashion to strokes, which can be a life-threatening diagnosis with serious implications if missed. The diagnosis is typically a clinical diagnosis, and the need for further testing is not necessary. Further testing is indicated in select cases and can be done with neuroimaging, cerebrospinal fluid analysis, and blood work if there are other neurological findings, cases of recurrent Bell Palsy, concerns for Lyme disease or other infectious etiology, or if an autoimmune etiology is suspected [11]. Given the low likelihood of alternative etiologies in this case, further lab testing and imaging beyond blood glucose measurement were not done in our patient.

Response to treatment is another important topic to address. In our case, the mother reported that she felt the patient had a 90% reduction in symptoms two months following treatment. She also stated that the patient’s father still had residual symptoms many years after he was initially diagnosed. Response to treatment can vary among patients. In patients who are not treated with steroids, roughly 70% of patients recover spontaneously by 3-6 months. That percentage increases to 80-85% if patients are treated with steroids [12].

This case poses the question of whether or not there may be a familial component that may predispose patients to the development of Bell’s palsy. A firm hereditary basis has yet to be discovered, but there are multiple prior reports showing evidence of familial incidences [13]. Mother stated that dad had Bell’s palsy at a very young age and still has residual symptoms, and that the patient’s aunt had Bell’s palsy, but could not tell me an exact age.

## Conclusions

Idiopathic facial nerve palsy (Bell's palsy) is a very uncommon illness in children and infants, with a clear cause and treatment still not optimally identified. In this case, a child with facial nerve palsy without an identifiable genetic, infectious, or traumatic illness is described. We propose that this is secondary to familial facial nerve palsy, as there is a clear history in the father and other family members of experiencing facial nerve palsy at a similar age. This patient was given steroids for treatment and ocular lubricant following the recommendations for adults. Further study should be done to determine the optimal treatment for idiopathic facial nerve palsy in children and infants.
